# Deficiency of p110δ Isoform of the Phosphoinositide 3 Kinase Leads to Enhanced Resistance to *Leishmania donovani*

**DOI:** 10.1371/journal.pntd.0002951

**Published:** 2014-06-19

**Authors:** Forough Khadem, Zhirong Mou, Dong Liu, Sanjay Varikuti, Abhay Satoskar, Jude E. Uzonna

**Affiliations:** 1 Department of Immunology, Faculty of Medicine, University of Manitoba, Winnipeg, Manitoba, Canada; 2 Department of Pathology, Ohio State University, Columbus, Ohio, United States of America; Queensland Institute of Medical Research, Australia

## Abstract

**Background:**

Visceral leishmaniasis is the most clinically relevant and dangerous form of human leishmaniasis. Most traditional drugs for treatment of leishmaniasis are toxic, possess many adverse reactions and drug resistance is emerging. Therefore, there is urgent need for identification of new therapeutic targets. Recently, we found that mice with an inactivating knock-in mutation in the p110δ isoform of pi3k, (p110δ^d910a^) are hyper resistant to *L. major*, develop minimal cutaneous lesion and rapidly clear their parasite. Here, we investigated whether pi3k signaling also regulates resistance to *L. donovani*, one of the causative agents of visceral leishmaniasis.

**Methodology/Principal Findings:**

WT and p110δ^D910A^ mice (on a BALB/c background) were infected with *L. donovani*. At different time points, parasite burden and granuloma formation were assessed. T and B cell responses in the liver and spleen were determined. In addition, Tregs were expanded *in vivo* and its impact on resistance was assessed. We found that p110δ^D910A^ mice had significantly reduced splenomegaly and hepatomegaly and these organs harbored significantly fewer parasites than those of WT mice. Interestingly, infected p110δ^D910A^ mice liver contains fewer and less organized granulomas than their infected WT counterparts. Cells from p110δ^D910A^ mice were significantly impaired in their ability to produce cytokines compared to WT mice. The percentage and absolute numbers of Tregs in infected p110δ^D910A^ mice were lower than those in WT mice throughout the course of infection. *In vivo* expansion of Tregs in infected p110δ^D910A^ mice abolished their enhanced resistance to *L. donovani* infection.

**Conclusions/Significance:**

Our results indicate that the enhanced resistance of p110δ^D910A^ mice to *L. donovani* infection is due to impaired activities of Tregs. They further show that resistance to *Leishmania* in the absence of p110δ signaling is independent of parasite species, suggesting that targeting the PI3K signaling pathway may be useful for treatment of both visceral and cutaneous leishmaniasis.

## Author Summary

Visceral leishmaniasis (VL) is the most dangerous form of human leishmaniasis in terms of mortality and morbidity and is spreading to several non-endemic areas because of global traveling and military conflicts. The emergence of *Leishmania*-HIV coinfection and increased prevalence of drug resistant strains have compounded an already bad situation. In addition, the drugs available are toxic, expensive and have several side effects. Therefore, a detailed understanding of protective immune response is extremely important in order to identify new therapeutic targets. The phosphoinositide 3 kinase (PI3K) family of enzymes mediate several important immunologic and physiologic cellular process including proliferation, differentiation, growth and host defense. We previously showed that genetic inactivation of the p110δ isoform of PI3K results in resistant to *L. major* (the causative agent of cutaneous leishmaniasis (CL)). Here, we investigate the role of PI3K in immunity to VL and the mechanisms underlying its protective effect. Collectively, our results demonstrate that signaling via the p110δ also regulates immunity to *L. donovani*, an effect that is dependent on the impact of p110δ signaling on expansion and function of regulatory T cells *in vivo*. Thus, our studies suggest that targeting the p110δ pathway may be a novel therapeutic strategy for controlling VL and CL.

## Introduction

Leishmaniasis is a vector borne disease that spreads through the bite of infected female sand fly [1]. An estimated 10–15 million cases of leishmaniasis occur worldwide in 98 tropical/subtropical countries [2], [3]. The disease is spreading to several non-endemic areas of the world and Leishmania-HIV coinfection has become increasingly problematic [4]. Leishmaniasis typically presents as one of the three forms, cutaneous (CL), mucocutaneous (ML) and visceral leishmaniasis (VL) [5], [6]. VL is caused by *L. donovani*, *L. infantum* (syn *L. chagasi*) in the Old World and by *L. chagasi* in the New World [7]. The estimated annual global burden of VL is about 200,000–400,000 new cases, and it remains the most important clinical form of the disease in humans in terms of mortality and morbidity [2]. Therefore, there is an urgent need to develop new drugs or vaccines that are non-toxic, cheap and effective.

The overall clinical symptoms, resistance and susceptibility to VL depend on several factors including the strain and specie of *Leishmania* and the nature of the host immune response [8], e.g. whether it is associated with the production of macrophage-activating cytokines such as Interferon-γ (IFN-γ) and Tumor Necrosis Factor-α (TNF-α) or macrophage-deactivating cytokines such as Interleukin-10 (IL-10) and Transforming Growth Factor-β (TGF-β) [4]. In general, susceptibility to *L. donovani* infection is mainly correlated with increased IL-10 production in humans [9] as well as in mice [10]. Both CD4^+^ and CD8^+^ T cells contribute to optimal protection against experimental *L. donovani* infection [11] by either regulating tissue damage or promoting parasite replication [12].

Regulatory T cells (Tregs), which are CD4^+^ T cells that express CD25 and Foxp3, play important role in immune regulation and homeostasis by suppressing several pathological and physiological immune responses [13]. Although Tregs primarily maintain self-tolerance and prevent autoimmunity, they also contribute to the pathogenesis of several infectious diseases including CL [14], [15]. Several types of Tregs exist, some of which are induced in response to infectious challenge while others are naturally endowed with regulatory properties (so called natural Tregs) [16]. Although natural Tregs consist of only 5–10% of peripheral CD4^+^ T cells in normal rodents and humans, they have potent effects on the activity of both CD4^+^ and CD8^+^ T cells by producing immunoregulatory cytokines, such as IL-10 and TGF-β [15]. Tregs have been shown to play a critical role in determining the outcome of *Leishmania* infection in mice [17] and humans [18]. For example, Foxp3^+^ cells accumulate at the pathologic sites of infection and play a role in both murine [17] and human VL [18]. Furthermore, a recent study showed that injection of IFN-γ inducible protein (CXCL10/IP-10) into *L. donovani*-infected mice causes a decrease in IL-10 and TFG-β production and this was correlated with reduction in numbers of CD4^+^CD25^+^ Tregs [19]. In addition, CD4^+^Foxp3^+^ Tregs accumulate in the vicinity of hepatic granulomas and this was associated with increased IL-10 mRNA and parasite persistence during VL in immunodeficient mice [17]. In contrast to these reports, Nyelen et al [9], reported that CD4^+^Foxp3^−^ cells were the major producers of IL-10 in human VL.

The class IA phosphoinositide 3-kinases (PI3Ks) are a family of lipid kinases that control multiple cellular processes including cell differentiation, growth, proliferation, migration, metabolism, survival [20] and immune response [21], [22]. Mammals have 3 catalytic subunits of class IA PI3Ks [20], [23] with the p110δ isoform being highly enriched in leukocytes [24]. The p110δ isoform seems to be adapted to transmit antigen-receptor signaling in T cells [20]. Indeed, naive CD4^+^ T cells from mice with an inactivating knock-in mutation in the p110δ gene, known as p110δ^D910A^, proliferated poorly and produce significantly less cytokines than cells from wild-type mice [25]. Interestingly, we found that p110δ^D910A^ mice were hyper-resistance to *L. major* (the causative agent of CL), develop minimal or no cutaneous lesion and rapidly clear their parasite despite mounting suppressed Th1 and Th2 responses [26]. This enhanced resistance was independent of mouse genetic background and was associated with dramatic amelioration of inflammatory response and decreased numbers and function of Tregs. Whether this pathway also controls resistance to *L. donovani*, the causative agent of VL is not known. Since regulation of host immunity to different *Leishmania* spp. may be highly variable, we investigated the outcome of infection of p110δ^D910A^ mice with *L. donovani* and the underlying mechanism(s) that regulate such disease outcome. We hypothesized that the p110δ isoform of PI3K pathway also controls disease outcome in mice infected with *L. donovani*. Consistent with this hypothesis, we show that deficiency of p110δ signaling results in hyper-resistance to experimental VL due in part to impaired Tregs activities, suggesting that targeting this pathway may be useful for treatment of the disease.

## Materials and Methods

### Mice

Female BALB/c mice were purchased from GMC, University of Manitoba. C57BL/6 (B6) mice that express an inactive form of p110δ isoform of PI3K (termed p110δ^D910A^) were generated by introducing a germline point mutation into the p110δ gene as previously described [27]. BALB/c p110δ^D910A^ mice were bred at the GMC facility of the University of Manitoba and were originally generated by backcrossing B6/129 p110δ^D910A^ mice onto the BALB/c background for more than 12 generations. All mice were maintained at the University of Manitoba Animal Care facility under specific pathogen-free conditions and used according to guidelines stipulated by the Canadian Council for Animal Care. The studies were approved by the University of Manitoba Animal Care and Use Committee (Protocol Approval number 12–072).

### Infection and parasite quantification

*Leishmania donovani* parasites (strain LV9) were grown in M199 insect culture medium (Invitrogen, Grand Island, NY) supplemented with 10% heat-inactivated FBS (HyClone, Logan, UT), 2 mM glutamine, 100 U/ml penicillin and 100 µg/ml streptomycin. Mice were injected with 5 × 10^7^ stationary phase promastigotes or 1 × 10^7^ amastigotes (isolated from spleen of 8–10 wks infected hamsters) in 100 µl PBS suspension intravenously (i.v.). Parasite burden in the spleen and liver was determined by limiting dilution assay [28].

### *In vitro* infection of bone marrow-derived macrophages (BMDMs)

Bone marrow cells were isolated from the femur and tibia of WT and p110δ^D910A^ mice. The cells were differentiated into macrophages (BMDMs) using complete medium supplemented with 30% L929 cell culture supernatant. BMDMs were harvested on day 7 and infected at a cell-to-parasite ratio of 1:5. After 5 hr of infection, free parasites were washed away and infected cells were further cultured for 24–72 hrs and the level of infection was determined by counting Giemsa-stained cytospin preparations under light microscope at ×100 (oil) objective.

### Isolation of splenic and hepatic cells and flow cytometry

At different days post infection, mice were sacrificed and infected spleen were homogenized in 10 ml DMEM media using tissue grinders and centrifuged at 1000 rpm for 5 min. Liver cells were also prepared as previously described with some minor modifications [29]. Briefly, liver cell suspensions were resuspended in 40% percoll, layered on top of 70% percoll and centrifuged at 750 g for 20 min at 22°C. After centrifugation, the interface layer containing lymphocytes was harvested and washed twice in complete DMEM medium (DMEM supplemented with 10% heat-inactivated FBS, 2 mM glutamine, 100 U/ml penicillin, and 100 µg/ml streptomycin). The liver and spleen cells were directly stained *ex vivo* for CD3, CD4, CD8, CD25 (extracellular staining) and Foxp3 (intracellular staining using BD Biosciences Foxp3 Staining Kit) expression for phenotypic flow cytometry analyses. In some experiments, liver and spleen cells were also directly stained *ex vivo* for intracellular cytokine analysis as previously described [26]. Briefly, cells were stimulated with 50 ng/ml PMA, 500 ng/ml ionomycin, and 10 µg/ml Brefeldin A for 4 hrs, fixed, surface-stained with specific fluorochrome-conjugated mAbs against CD3, CD4 and CD8 and stained intracellularly for IFN-γ, IL-4 and IL-10. Samples were acquired on a FACSCanto II cytometer (BD Bioscience, San Diego, CA) and analyzed using Flowjo software (Tree Star, Ashland, OR).

### *In vivo* expansion of Tregs

Tregs were selectively expanded *in vivo* by injecting mice with IL-2-anti-IL-2 mAb immune complexes according to recently published reports [30], [31] with some adjustments. Briefly, rIL-2 (PeproTech, Rocky Hill, NJ) was mixed with anti-IL-2 mAb (clone JES6-1, BD Bioscience) and incubated at 37°C for 30 min. Wild type and p110δ^D910A^ mice were injected intraperitoneally (i.p.) with the immune complex containing 1 µg rIL-2 and 5 µg anti-IL-2 mAb once a day for 3 days. Three days after the last injection, mice were infected with 5 × 10^7^ stationary phase *L. donovani* promastigotes. Thereafter, the immune complex was administrated once weekly until mice were sacrificed.

### *In vitro* recall responses and cytokine ELISA

Single cell suspensions of cells from the liver and spleen of infected mice were resuspended at 4 × 10^6^/ml in complete DMEM medium, plated at 1 ml/well in 24-well tissue culture plates and stimulated with freeze thawed *L. donovani* (10 µg/ml). After 72 hr, the supernatant fluids were collected and assayed for cytokines (IL-4, IL-12, IL-10 and IFN-γ) by ELISA using paired antibodies (Biolegend, San Diego, CA) according to manufacturer's suggested protocols. In some cases, the cytokine levels were determined by Flowcytomix array using reagents from BD Biosciences.

### Measurement of serum antibody levels and NO assay

At sacrifice, serum was obtained from infected mice and used to determine the levels of anti-*Leishmania*-specific antibody titers (IgG, IgM, IgG1 and IgG2a) by ELISA as previously described [32]. NO levels were determined by measuring nitrite concentration in the culture supernatant fluids using the Griess assay [33].

### Assessment of hepatic granuloma

The granulomatous response to infection in the liver was assessed in histologic sections stained with hematoxylin and eosin at 2, 4 and 8 weeks post infection as described elsewhere [34], [35]. At each time point, sections from at least 6 individual mice were analyzed in each group. Granuloma formation was scored as follows: ineffective granulomas, large quantities of mononuclear cells forming adjacent to sinusoids with no mononuclear cell infiltration to the tissue; developing granulomas, some functional mononuclear cellular infiltration at the parasitized focus; and mature granulomas, a core of functional fused infected Kupffer cells surrounded by a well-developed epithelioid-type mononuclear cell mantle.

### Statistical analysis

A two way ANOVA was used to analyze the results. Results are representative of 2 to 4 independent experiments (n  =  3–4 mice per group) with similar results. Error bars indicate +/– SEM and data were considered significant when p < 0.05.

## Results

### Mice with inactive p110δ PI3K are highly resistant to *L. donovani* infection

**Figure 1:**
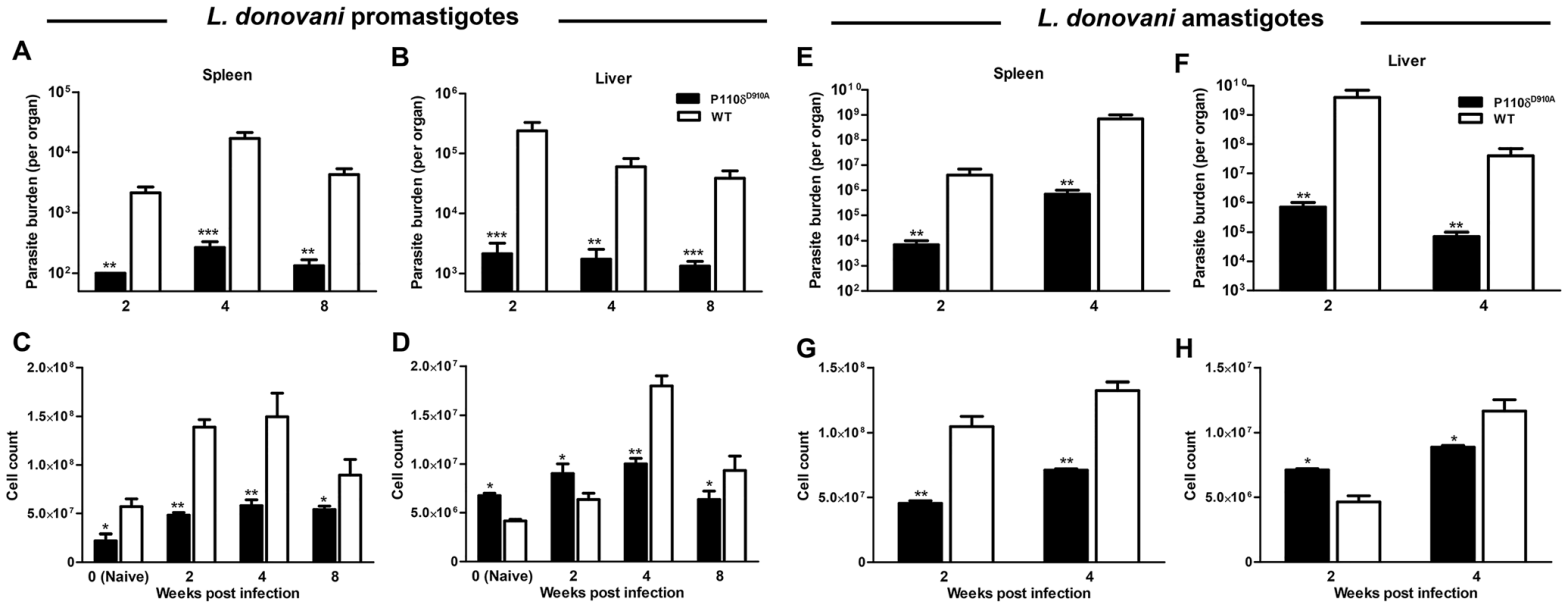
P110δ^D910A^ mice are hyper-resistant to *L. donovani*. (A, B) Kinetics of parasite burden in the spleens and liver of WT and p110δ^D910A^ BALB/c mice. Mice were infected with 5 × 10^7^ stationary phase promastigotes (A, B) or 1 × 10^7^ hamster spleen-derived amastigotes (E, F) and sacrificed at different times (as indicated) to assess parasite burden in the spleens (A, E) and liver (B, F). Total number of cells in the spleens (C, G) and liver (D, H) of WT and p110δ^D910A^ mice at different times post-infection with promastigotes (C, D) or amastigotes (G, H). Results are representative of 6 (A–D) and 2 (E–H) independent experiments (n  =  4 mice per group) with similar results. Error bars, +/− SEM; *, p < 0.05; **, p < 0.01; ***, p < 0.001.

We previously showed that despite significantly impaired T cell responses, p110δ^D910A^ mice are highly resistant to *L. major*, the causative agent of CL [26]. To determine whether signaling via the p110δ isoform of PI3K also regulates resistance to VL, we infected WT and p110δ^D910A^ mice intravenously with *L. donovani* promastigotes or amastigotes at different times after infection, assessed parasite burden in the spleens and liver by limiting dilution assay. In agreement with our previous observation with *L. major* [26], *L. donovani*-infected p110δ^D910A^ mice were more resistant than their WT counterparts. By two weeks post-infection, p110δ^D910A^ mice harbored significantly fewer parasites than infected WT mice both in their spleens (Figure 1A and 1E, p < 0.01) and livers (Figure 1B and 1F, p < 0.001) and this trend was maintained for several weeks (up to 8 weeks post-infection). Consistent with this reduced parasite burden, the spleens and livers of infected p110δ^D910A^ mice were significantly smaller than WT mice, indicating that hepatomegaly and splenomegaly, which are marked features of VL, were significantly controlled in *L. donovani* infected p110δ^D910A^ mice (Figure S1). The reduction in splenic and hepatic sizes in infected p110δ^D910A^ mice was correlated with significantly reduced numbers of cells in these organs (Figure 1C–1D and 1G–1H), suggesting that deficiency of p110δ might affect cellularity and/or increased cell proliferation or recruitment into these organs.

Because *L. donovani* is known to activate PI3K/AKT in macrophages [36], which might influence parasite replication, we determined whether the enhanced resistance of p110δ^D910A^ mice was related to hyperactivity of their macrophages in restricting parasite growth. Both WT and p110δ^D910A^ BMDMs were equally permissive to *L. donovani* following *in vitro* infection (Figure S2), suggesting that as reported previously for *L. major* [26], the enhanced resistance of p110δ^D910A^ mice to *L. donovani* is not due to enhanced responsiveness or leishmaniacidal activities of their macrophages.

### Splenic and hepatic immune (cytokine) responses in *L. donovani*-infected p110δ^D910A^ mice

The observation of enhanced resistance (lower parasite burden) in p110δ^D910A^ mice following *Leishmania* infection, prompted us to assess their T cell responses. Infected p110δ^D910A^ mice had fewer leukocytes than WT mice in the spleens during the course of infection (Figure 1C and 1G). Surprisingly, in the liver, the leukocyte count was slightly higher in the p110δ^D910A^ mice at 2 weeks post-infection and significantly lower at 4 and 8 weeks post infection compared to WT infected mice (Figure 1D and 1H).

**Figure 2:**
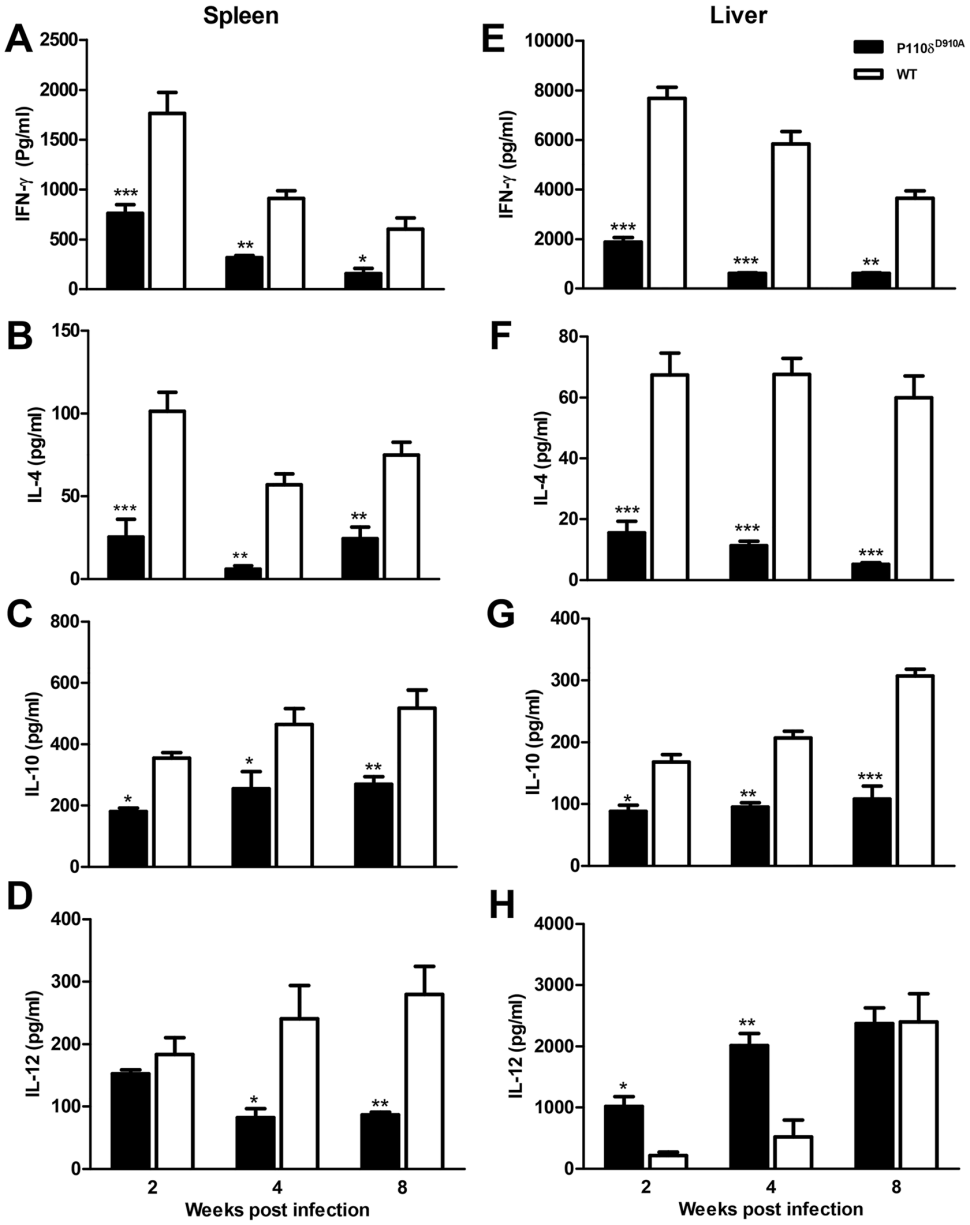
Impaired cytokine production by spleen and liver lymphocytes from *L. donovani*-infected highly resistant p110δ^D910A^ mice. At the indicated times after infection, spleen and liver lymphocytes from WT and p110δ^D910A^ mice were cultured *in vitro* in the presence of *L. donovani* antigen for 72 hrs and the culture supernatant fluids were assayed for cytokines by Flowcytomix array. Shown are the splenic values for IFN-γ (A), IL-4 (B), IL-10 (C) and IL-12 (D) and liver values for IFN-γ (E), IL-4 (F), IL-10 (G) and IL-12 (H) at different times post-infection. Results are representative of 3 independent experiments (n  =  4 mice per group) with similar results. Error bars, +/− SEM; *, p < 0.05; **, p < 0.01; ***, p < 0.001; ND, Not Detected.

To determine whether the enhanced resistance of p110δ^D910A^ mice was associated with superior effector cellular cytokine response, we assessed splenic and hepatic cells from infected mice for their cytokine response directly *ex vivo* (by flow cytometry) or after 3 days restimulation *in vitro* with *L. donovani* antigen by ELISA. At all time points after infection, the percentages and absolute numbers of IFN-γ-producing (Figure S3) and IL-4-producing (Figure S4) cells in the spleens and livers of infected highly resistant p110δ^D910A^ mice were significantly lower than those from their infected WT counterpart mice. Interestingly, while CD4^+^ cells were the major producers of IFN-γ in both organs, IL-4 producing cells were mostly from CD3^−^ lymphocyte population (Figure S4). Consistent with the flow data, splenic and hepatic lymphocytes from infected p110δ^D910A^ mice also produced significantly less IFN-γ, IL-4, IL-10 and TNF in culture supernatant fluids compared to those from WT mice (Figure 2A-G and data not shown). Interestingly, while spleen cells from p110δ^D910A^ mice produced significantly less IL-12 in cultures compared to WT mice, their hepatic cells produced more of this cytokine than those from WT mice (Figure 2D and 2H). Similarly, while the levels of nitric oxide (NO), key effector molecule for killing *Leishmania* inside infected cells, were significantly lower in the spleen cell cultures from infected p110δ^D910A^ mice, they were comparable in cultures from liver cells from infected p110δ^D910A^ and WT mice (Figure S5). Collectively, these findings show that the loss of p110δ activity is sufficient to reverse the susceptibility of infected BALB/c mice to *L. donovani* infection despite having impaired cytokine responses.

### Impaired antibody response in *L. donovani* infected p110δ^D910A^ mice

**Figure 3:**
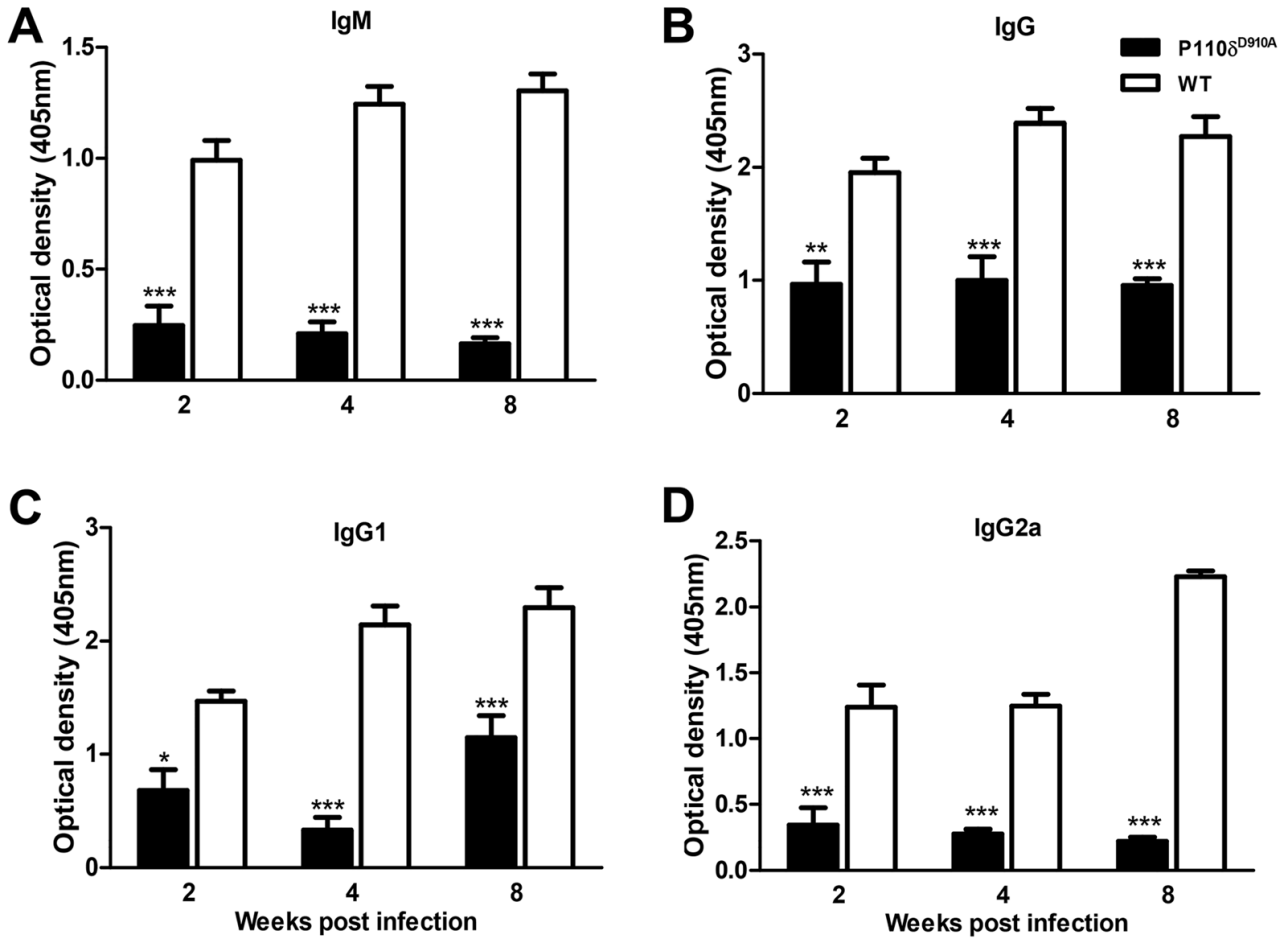
Impaired antibody response in resistant p110δ^D910A^ mice. Total antigen-specific IgM (A), IgG (B), IgG1 (C) and IgG2a (D) levels in the sera of infected p110δ^D910A^ and WT mice. At different times after infection, mice were sacrificed and sera were analyzed for different isotypes of *Leishmania*-specific antibodies by ELISA. Results are representative of 3 independent experiments (n  =  4 mice per group) with similar results. Error bars, +/− SEM; *, p < 0.05; **, p < 0.01; ***, p < 0.001.

Previous reports show that p110δ^D910A^ mice have reduced numbers of peripheral B cells as well as impaired B cell signaling and a concomitant reduction in circulating plasma cells and serum antibody levels [27], [37], [38]. In addition, we previously found that the total IgG as well as parasite-specific IgG1 and IgG2a levels in the sera of *L. major*-infected p110δ^D910A^ mice were significantly lower than in WT controls [26]. Therefore we assessed whether infection with *L. donovani* was also associated with impaired B cell responses. As shown in Figure 3A–D, the parasite-specific IgG and IgM as well as IgG1 and IgG2a levels in the sera of *L. donovani*-infected p110δ^D910A^ mice were significantly lower than in WT controls during the course of infection. The significantly lower antibody response was not responsible for the enhanced resistance of p110δ^D910A^ mice to *L. donovani* because injection of serum from *L. donovani*-infected WT mice (which contains high levels of *L. donovani*-specific IgG) did not abolish the enhanced resistance of p110δ^D910A^ mice to the parasite (data not shown). Collectively, these results indicate that as observed in *L. major* infection [26], impaired B cell response and/or antibody production is not responsible for the enhanced resistance of p110δ^D910A^ to *L. donovani*.

### Impaired granuloma formation in *L. donovani*-infected p110δ^D910A^ mice

**Figure 4:**
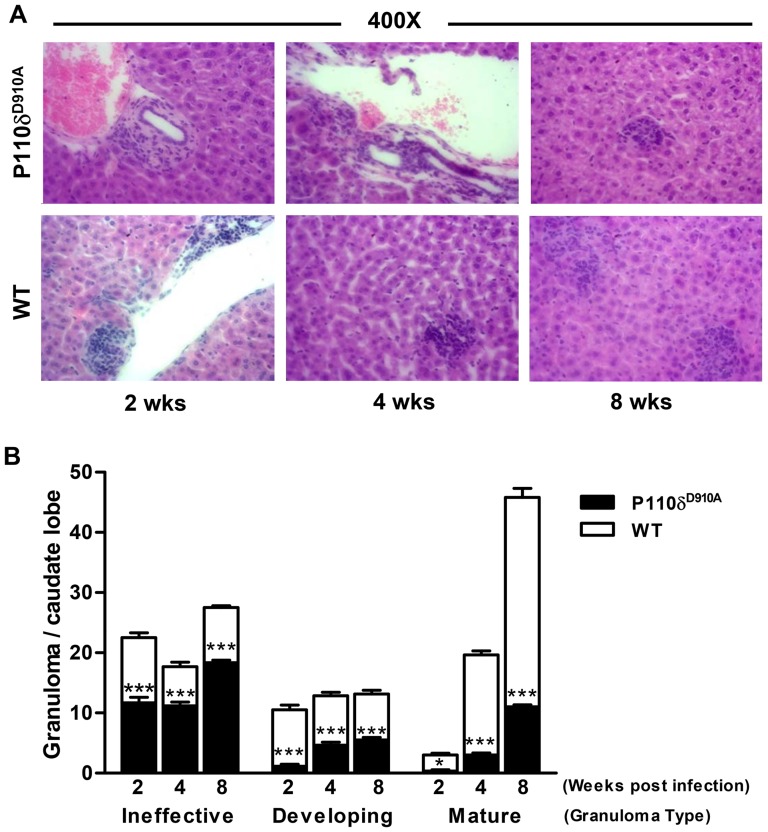
Enhanced resistance of p110δ^D910A^ mice is not associated with more robust granuloma formation. Infected p110δ^D910A^ and WT mice were sacrificed at the indicated times and their liver were processed and stained routinely to assess granuloma formation (size, cellularity and maturation) as described in the materials and methods section. The H&E stained sections (A) were assessed and scored blindly by a pathologist for the presence/number of ineffective, developing and mature granulomas and represented as a bar graph (B). Results are representative of 2 independent experiments (n  =  3 mice per group) with similar results. Error bars, +/− SEM; *, p < 0.05; **, p < 0.01; ***, p < 0.001.

*Leishmania*-specific immune response in the liver leads to the formation of granulomas that limit infection, kill and remove the microbial target and repair any accompanying tissue injury [35]. Enhanced resistance to *L. donovani* infection in mice has been linked to formation of effective granuloma [39]–[41]. Because p110δ^D910A^ mice are strongly resistant to *L. donovani*, we hypothesized that this would be linked to more efficient and effective granuloma formation in their livers. Therefore, we assessed granuloma formation in H&E sections in these organs at different times after infection. By week 2 post-infection in WT mice, mononuclear cells were recruited to adjacent sinusoids and ineffective granulomas with no mononuclear cell infiltration were already formed. In addition, developing functional granulomas were starting to generate by parasitized Kupffer cells fusing together and this was surrounded by foci of infiltrating lymphocytes and monocytes. By week 4 post-infection, developing and/or mature granulomas were visible and involuting large epithelioid granuloma devoid of amastigotes were clearly present by week 8 post-infection (Figure 4A and 4B). In contrast, mostly ineffective granulomas and only very few developing functional granulomas were visible in tissues from infected p110δ^D910A^ mice by 4 weeks post-infection such that by 8 weeks post-infection, mononuclear cells were still remaining largely within adjacent sinusoids and significantly fewer numbers of developing or smaller mature granulomas were present (Figure 4A and 4B). Thus, contrary to the established dogma, enhanced resistance to *L. donovani* infection in p110δ^D910A^ mice was not associated with more effective granuloma formation in the liver.

### Regulatory T cells in *L. donovani*-infected p110δ^D910A^ mice

**Figure 5:**
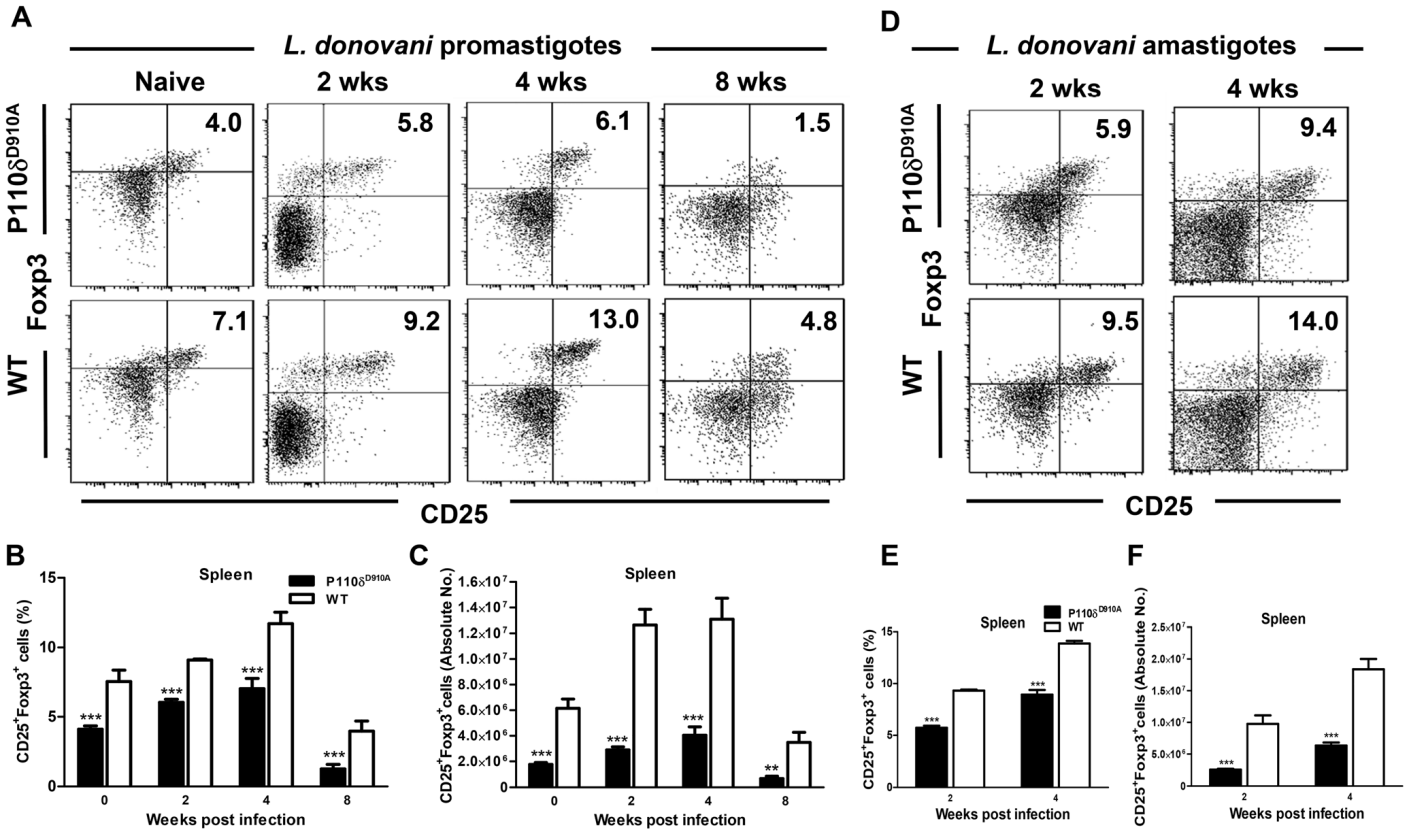
Reduced number of CD4^+^CD25^+^Foxp3^+^ T cells (Tregs) in p110δ^D910A^ mice. Flow cytometry showing the percentages (A, B) and absolute numbers (C) of CD4^+^CD25^+^Foxp3^+^ (Tregs) in the spleens of WT and p110δ^D910A^ mice infected with *L. donovani* promastigotes at different times post-infection. The percentages (D, E) and absolute numbers (F) of Tregs in the spleens of WT and p110δ^D910A^ mice infected with *L. donovani* amastigotes were also assessed. Splenocytes of uninfected (naïve) and infected mice were directly stained *ex vivo* for CD3, CD4, CD25 and Foxp3 at 2, 4 and 8 weeks post-infection. Representative dot plots (A, D) and bar graphs showing the mean +/− SEM of the percentages (B, E) and absolute numbers (C, F) of CD25^+^Foxp3^+^ cells are shown after gating on CD3^+^CD4^+^ population. Results are representative of 3 independent experiments (n  =  4 mice per group) with similar results. Error bars, +/− SEM; *, p < 0.05; **, p < 0.01; ***, p < 0.001.

Tregs contribute to susceptibility to *L. donovani* infection [42], [43], in part by enhancing parasite persistence in infected organs [17]. In addition, previous reports show that p110δ^D910A^ mice have impaired expansion of Tregs [27], [44] and this was in part responsible for their enhanced resistance to *L. major* [26]. To determine whether the enhanced resistance of p110δ^D910A^ mice to *L. donovani* is related to impaired induction and/or expansion of Tregs, we compared the percentage (Figure 5A, 5B, 5D and 5E) and absolute numbers (Figure 5C and 5F) of CD4^+^CD25^+^Foxp3^+^ cells (Tregs) in the spleens of *L. donovani*-infected p110δ^D910A^ and WT mice. At all times tested, the percentages and absolute numbers of Tregs in the spleens of infected p110δ^D910A^ mice were significantly lower than in their WT counterpart mice. The data also show that in both WT and p110δ^D910A^ mice, infection with *L. donovani* leads to increase in the number of Tregs, peaking around week 4 and returning to baseline by week 8 post-infection. However, this increase was significantly higher in WT than in p110δ^D910A^ mice. Interestingly, most of the CD25^+^ T cells in infected mice also co-expressed Foxp3, suggesting that during *L. donovani* infection, most of activated CD25^+^ T cells are skewed towards a Treg phenotype. Taking together, these results suggest that impaired expansion and/or function of Tregs may be responsible for the enhanced resistance of p110δ^D910A^ mice to *L. donovani* infection.

### Systemic *in vivo* expansion of Tregs renders p110δ^D910A^ mice susceptible to *L. donovani* infection

**Figure 6:**
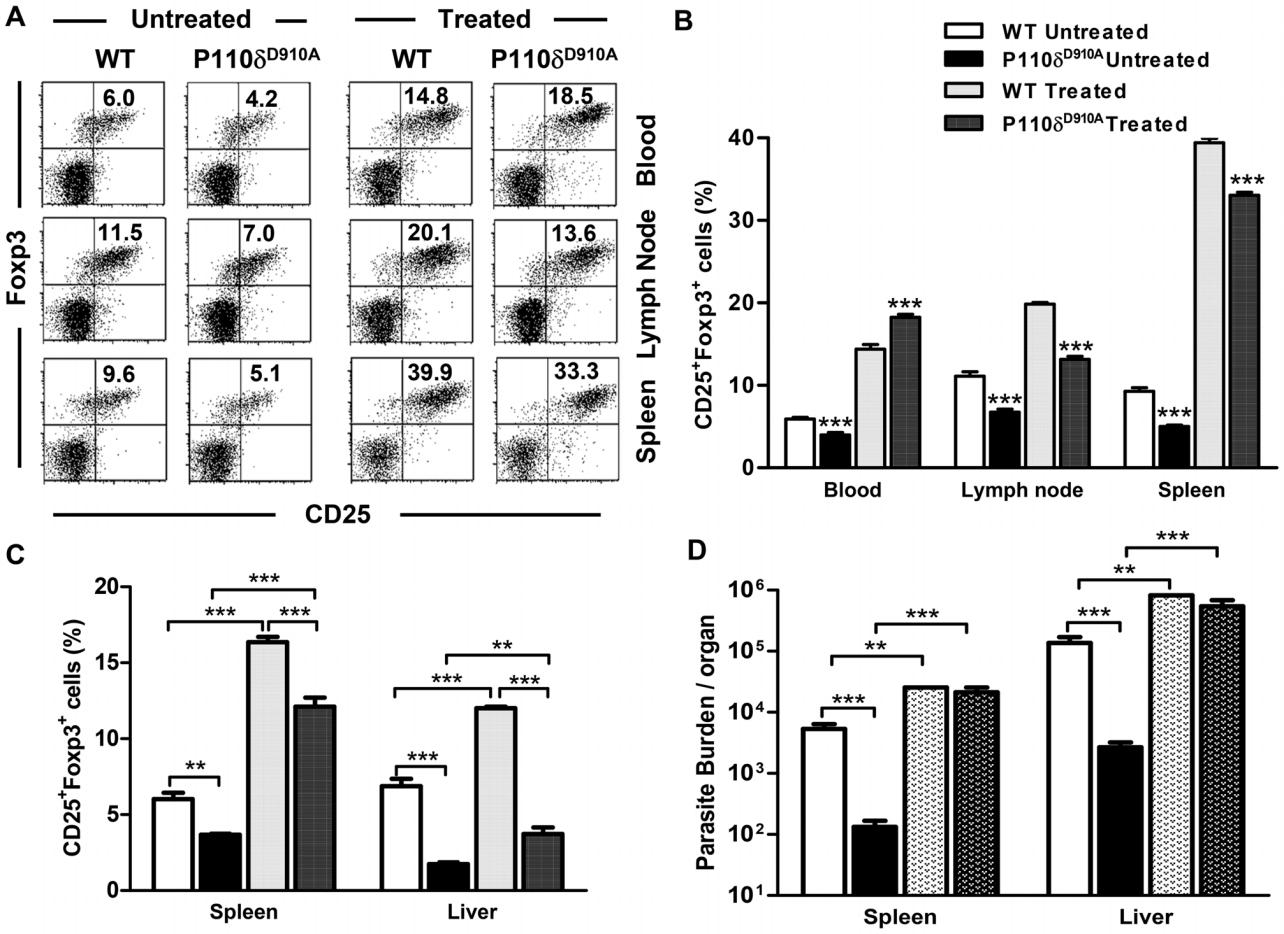
Systemic Treg expansion by administration of IL-2/anti-IL-2 immune complex leads to abrogation of enhanced resistance to *L. donovani* in p110δ^D910A^ mice. WT and p110δ^D910A^ mice were injected intraperitoneally with rIL-2/anti-IL-2 mAb immune complex (treated) once a day for three consecutive days. Control mice were injected with isotype-matched control antibody mixed with rIL-2 (untreated). Two days after the last immune complex injection, mice were sacrificed and the percentage of CD4^+^CD25^+^Foxp3^+^ cells (Tregs) in the blood, lymph nodes and spleens was determined directly *ex vivo*. Representative dot plots (A) and bar graphs showing the mean +/− SEM of the percentages (B) of CD4^+^CD25^+^Foxp3^+^ cells in the blood, lymph nodes and spleens. In a different experiment, immune complex-treated (or untreated) mice were infected with 5 × 10^7^ *L. donovani* and immune complex treatment was continued once a week for 2 additional weeks. Infected mice were then sacrificed and the percentages of CD4^+^CD25^+^Foxp3^+^ cells (Tregs) in spleens and liver tissues were assessed directly *ex vivo* by flow cytometry (C). At sacrifice, parasite burden in the spleens and livers was assessed by limiting dilution assay (D). Results are representative of 2 independent experiments (n  =  4 mice per group) with similar results. Error bars, +/− SEM; *, p < 0.05; **, p < 0.01; ***, p < 0.001.

We speculated that the significantly lower numbers of Tregs after infection dampen Treg-mediated suppression of parasite killing leading to rapid clearance of parasites in infected p110δ^D910A^ mice despite lower T cell response. Therefore, we hypothesized that increasing Treg numbers in infected p110δ^D910A^ mice would abolish their enhanced resistance to *L. donovani*. To test this hypothesis, we utilized a novel *in vivo* approach for inducing rapid expansion of Tregs by injecting rIL-2/anti-IL-2 immune complex into naïve and infected mice. Consistent with previous reports [30], [31], this protocol led to rapid and comparable increase in the percentage and absolute numbers of Tregs in the spleen, liver, lymph node and blood of both uninfected (Figure 6A and B) and infected (Figure 6C) WT and p110δ^D910A^ mice, suggesting that Tregs have the ability to expand in p110δ^D910A^ mice.

Next, we infected WT and p110δ^D910A^ mice injected with rIL-2/anti-IL-2 immune complex with *L. donovani* and followed up with weekly injection of immune complex to maintain high levels of Tregs. Strikingly, expansion of Tregs results in dramatic abrogation of enhanced resistance of p110δ^D910A^ mice to *L. donovani* such that parasite burdens in the spleens and liver were significantly increased and indistinguishable from those of WT mice at 2 (Figure 6D) and 4 weeks (data not shown) post-infection. Collectively, these results show that the enhanced resistance to *L. donovani* is related to the significantly reduced numbers of Tregs in absence of p110δ signaling.

## Discussion

Leishmaniasis remains a global health problem and an understanding of the mechanisms that underlie host resistance and/or susceptibility to the disease could significantly impact on the development of new drugs and vaccines for human use. While *L. donovani* infection results in the development of some levels of immunity in the spleen, liver and bone marrow, the quality of this immunity is variable among organs and the exact immunologic and protective correlates of immunity remain poorly understood. For example, while infection in the liver is effectively controlled, *L. donovani* infection in the spleen remains chronic for months with no discernable immunologic defects in the infected mice. Understanding the mechanisms governing this organ-specific immunity is vital for effective therapeutic interventions against VL.

Members of the class 1A family of PI3K are important enzymes that control several important cellular events including cell differentiation, growth, proliferation and immune response [21], [22], and have been shown to regulate immunity to many pathogens including parasites [45], [46]. Infection of macrophages with *Leishmania* parasites results in engagement and sustained activation of the PI3K/Akt signaling pathway [47]. Unlike other isoforms of PI3K, which is expressed by many cell types, the p110δ isoform is mostly restricted to leucocytes including B cells, T cells and antigen presenting cells (macrophages and DCs) [48], suggesting that they may play critical role in immunity. *L. donovani* parasites engage TLR2 receptor on macrophages and induce mTOR signaling in PI3K-dependent and independent mechanisms [48]. Our previous studies highlight the importance of p110δ isoform of PI3K in the regulation of T cell-mediated immunity [26], [49]. We showed that p110δ^D910A^ mice, which exhibit attenuated Th1 responses, are protected against *L. major* infection even in the normally susceptible BALB/c background [26]. This finding challenges the Th1/Th2 paradigm as the primary determinant of resistance and susceptibility to Leishmaniasis, and instead focuses attention towards regulatory mechanisms that control inflammation as being key determinant of resistance and/or susceptibility.

In the present study, we further extend the importance of regulatory mechanisms that control inflammation in the pathogenesis of leishmaniasis by showing that p110δ^D910A^ mice are also highly resistant to *L. donovani*, the major *Leishmania* spp. that cause VL. We showed that in addition to having dramatically reduced splenic and hepatic parasite burdens in both promastigote and amastigote-initiated infections, hepatomegaly and splenomegaly (which are hallmarks of VL), were significantly controlled in *L. donovani* -infected p110δ^D910A^ mice. Importantly and consistent with the paradigm, the highly resistant p110δ^D910A^ mice presented impaired T cell responses by producing significantly less IFN-γ, IL-4, IL-10 and TNF levels both in the spleen and liver. Interestingly, *L. donovani* infection was also associated with impaired B cell (antibody) responses in these mice. However, passive transfer of immune serum from *L. donovani*-infected WT mice into p110δ^D910A^ mice did not abolish their enhanced resistance. This finding showed that the enhanced resistance of p110δ^D910A^ mice to *L. donovani* is not primarily related to their impaired B cell response, which is consistent with our previous observations in *L. major* infection [26].

Efficient and effective anti-*Leishmania* protection in the liver is usually achieved by granuloma formation around infected Kupffer cells. This is usually associated with chemokine production, recruitment of monocytes, neutrophils and T cells, production of inflammatory cytokines and activation of infected Kupffer cells. These events lead to the liver becoming an acute resolving site of the infection and resistant to reinfection. In contrast, although the spleen is the initial site for generating cell mediated-immune responses, it eventually becomes a site of parasite persistence with accompanying immunopathological changes and is associated with high levels of TNF and IL-10 [50]. Thus, it is believed that the formation of granuloma in the liver is beneficial to the host in restricting parasite proliferation [39]. Our results demonstrate that during the course of *L. donovani* infection, the livers of infected but highly resistant p110δ^D910A^ mice significantly contain fewer numbers of developing granulomas and smaller mature granulomas by 8 weeks post-infection. Thus, our results show that effective parasite control in the liver and enhanced resistance to *L. donovani* does not necessarily require granuloma formation. Granulomas are usually initiated to contain persistent pathogens and signal the presence of chronic inflammatory responses [39]. We speculate that granuloma formation may become necessary when there are regulatory mechanisms (such as in the presence of Tregs) that act to dampen effective T cell-mediated immunity. In the absence of such regulatory mechanisms (as in p110δ^D910A^ mice), high amounts of IFN-γ production is not needed for resistance, because the low IFN-γ response is very efficient at more effectively activating infected Kupffer cells leading to more efficient parasite destruction. In line with this, a recent report demonstrated the presence of Tregs in hepatic granulomas of *L. donovani*-infected mice and suggested that Tregs mediate parasite persistence and susceptibility to experimental VL caused by *L. donovani* [17]. However, it is conceivable that the reduced number of granulomas might be a consequence of rather than the cause of lower parasite burden in the liver of infected p110δ^D910A^ mice.

Our studies support the previous reports showing that Tregs contribute to the pathogenesis of experimental VL in mice [17], [43]. They further show that signaling via the p110δ isoform of PI3K is critical for functional competency of Tregs in mice. Despite having higher or similar numbers of Tregs in their thymus, p110δ^D910A^ mice have significantly lower numbers of CD4^+^CD25^+^ and CD4^+^CD25^-^ T cells in their peripheral tissues including lymph nodes and spleens [21] compared to WT mice. Consistent with this, we found that infected p110δ^D910A^ mice have significantly lower numbers of CD4^+^CD25^+^Foxp3^+^ (Tregs) in their spleens throughout the course of infection compared to their WT counterpart mice. Using *in vivo* Treg expansion strategy, we showed that the expansion of Tregs in naïve and infected WT and p110δ^D910A^ mice were comparable. Remarkably, this expansion of Tregs in p110δ^D910A^ mice completely abolished their enhanced resistance to *L. donovani* such that the parasite burden in the livers and spleens of infected p110δ^D910A^ and WT mice were comparable at all times after infection following *in vivo* Treg expansion (Fig. 6D). Thus, given appropriate stimulus, Tregs from p110δ^D910A^ mice are capable of expanding to a number that regulates anti-*Leishmania* immunity. This is consistent with our previous findings in *L. major* infection whereby adoptively transferring high numbers of p110δ^D910A^ Tregs back into p110δ^D910A^ mice was capable of abolishing the enhanced resistance to *L. major* infection akin to WT Tregs [26].

Collectively, our studies highlight the importance of the p110δ isoform of PI3K signaling pathway in regulating T cell-mediated immunity and suggest that targeting this pathway may have important and direct implications for immunomodulation and immunotherapy of VL. Due to several drawbacks associated with the current anti-*Leishmania* treatments, including prolonged duration of treatment, toxicity, high cost of treatment, emergence of drug resistance strains and disease relapse [5], [8], [12], efforts are being made to develop new drugs and treatment regimens. Given the dramatic hyper-resistance seen in p110δ^D910A^ mice infected with *L. donovani* and *L. major* [26], we speculate that the use of highly specific pharmacological inhibitors of p110δ may be beneficial in the treatment of human cutaneous and visceral leishmaniasis. Although these compounds are currently being developed for treatment of inflammatory conditions, it is likely they may also be beneficial in modulating immune response against leishmaniasis. Such immunomodulatory effects when combined with conventional therapy, may lower the required drug dose and treatment regimen, reduce drug toxicity, improve drug efficacy, reduce emergence of drug resistant strains and consequently reduce the chances of disease relapse.

## Supporting Information

Figure S1 **Reduced splenomegaly and hepatomegaly in infected p110δ^D910A^ mice.** WT and p110δ^D910A^ mice were infected with 5 × 10^7^ stationary phase promastigotes of *L. donovani*, sacrificed at 8 weeks post infection and the spleens (A) and livers (B) of infected mice were weighed. Results are representative of 3 independent experiments (n  =  4 mice per group) with similar results. Error bars, +/− SEM; *, p < 0.05; **, p < 0.01; ***, p < 0.001.
(TIF)

Figure S2 **Enhanced resistance of p110δ^D910A^ mice to** ***L. donovani*** **is not due to superior macrophage responsiveness.** Bone marrow-derived macrophages from WT and p110δ^D910A^ mice were infected with *L. donovani* promastigotes at a cell-to-parasite ratio of 1:5. After 24, 48 and 72 hrs, cytospin preparations were made, stained with Wright-Giemsa stain and the number of parasites per 100 macrophages (A), percent infectivity (B) and number of parasites per infected macrophages (C) were determined. (D) Light microscopy images (at ×100 (oil) objective) of infected macrophages in different time points. Results are representative of 2 independent experiments (n  =  3 mice per group) with similar results.
(TIF)

Figure S3 **Spleen and liver lymphocytes from infected resistant p110δ^D910A^ mice produce less IFN-γ than those from WT mice.** Spleen (A and B) and liver (C and D) lymphocytes from WT and p110δ^D910A^ mice infected with *L. donovani* amastigotes were assessed directly *ex vivo* at 2 and 4 weeks post infection for IFN-γ production by flow cytometry. Results are representative of 2 independent experiments (n  =  3 mice per group) with similar results. Error bars, +/− SEM; *, p < 0.05; **, p < 0.01; ***, p < 0.001.
(TIF)

Figure S4 **Non T cells (CD3^−^) are the major IL-4-producing cells in the spleens and liver of** ***L. donovani*** **infected WT and resistant p110δ^D910A^ mice.** *L. donovani* promastigote infected p110δ^D910A^ and WT mice were sacrificed at the indicated times and their spleen (A, B) and liver (C, D) lymphocytes were pulsed with PMA, ionomycin and brefeldin A (BFA) for 4 hrs and directly stained *ex vivo* for CD3, CD4 and IL-4. Results are representative of 3 independent experiments (n  =  3 mice per group) with similar results. Error bars, +/− SEM; *, p < 0.05; **, p < 0.01; ***, p < 0.001.
(TIF)

Figure S5 **Enhanced resistance of p110δ^D910A^ mice to** ***L. donovani*** **is not associated with high nitric oxide (NO) production.** NO levels were measured in 72 hr culture supernatant fluids of spleen (A) and liver (B) lymphocytes of *L. donovani*-infected WT and p110δ^D910A^ mice that were stimulated with freeze-thawed *L. donovani*. Results are representative of 3 independent experiments (n  =  3 mice per group) with similar results. Error bars, +/− SEM; *, p < 0.05; **, p < 0.01; ***, p < 0.001.
(TIF)
