# Supplementary figures and images for: Deficiency of p110δ Isoform of the Phosphoinositide 3 Kinase Leads to Enhanced Resistance to Leishmania donovani
Source: PLoS Negl Trop Dis. 2014 Jun 19;8(6):e2951. doi: 10.1371/journal.pntd.0002951 (PMC4063731; doi:10.1371/journal.pntd.0002951)

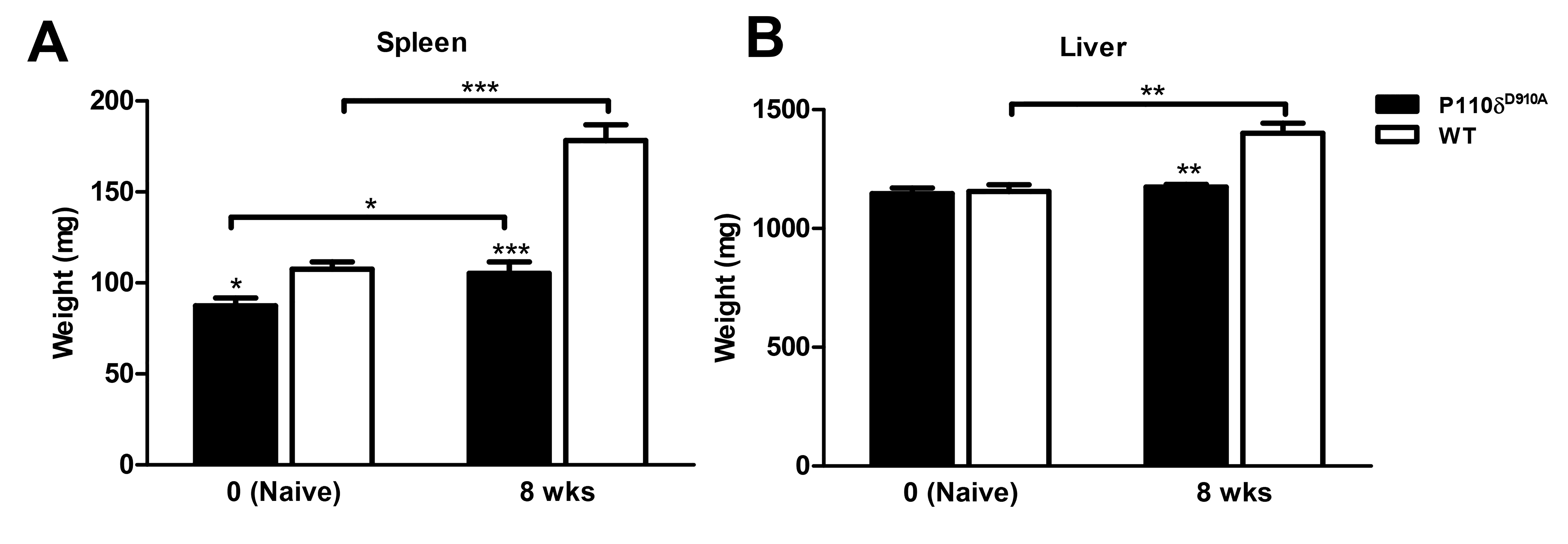

Supplement: Figure S1 — Reduced splenomegaly and hepatomegaly in infected p110δD910A mice. WT and p110δD910A mice were infected with 5 × 10^7^ stationary phase promastigotes of L. donovani, sacrificed at 8 weeks post infection and the spleens (A) and livers (B) of infected mice were weighed. Results are representative of 3 independent experiments (n = 4 mice per group) with similar results. Error bars, +/− SEM; *, p < 0.05; **, p < 0.01; ***, p < 0.001. (TIF) [file pntd.0002951.s001.tif]

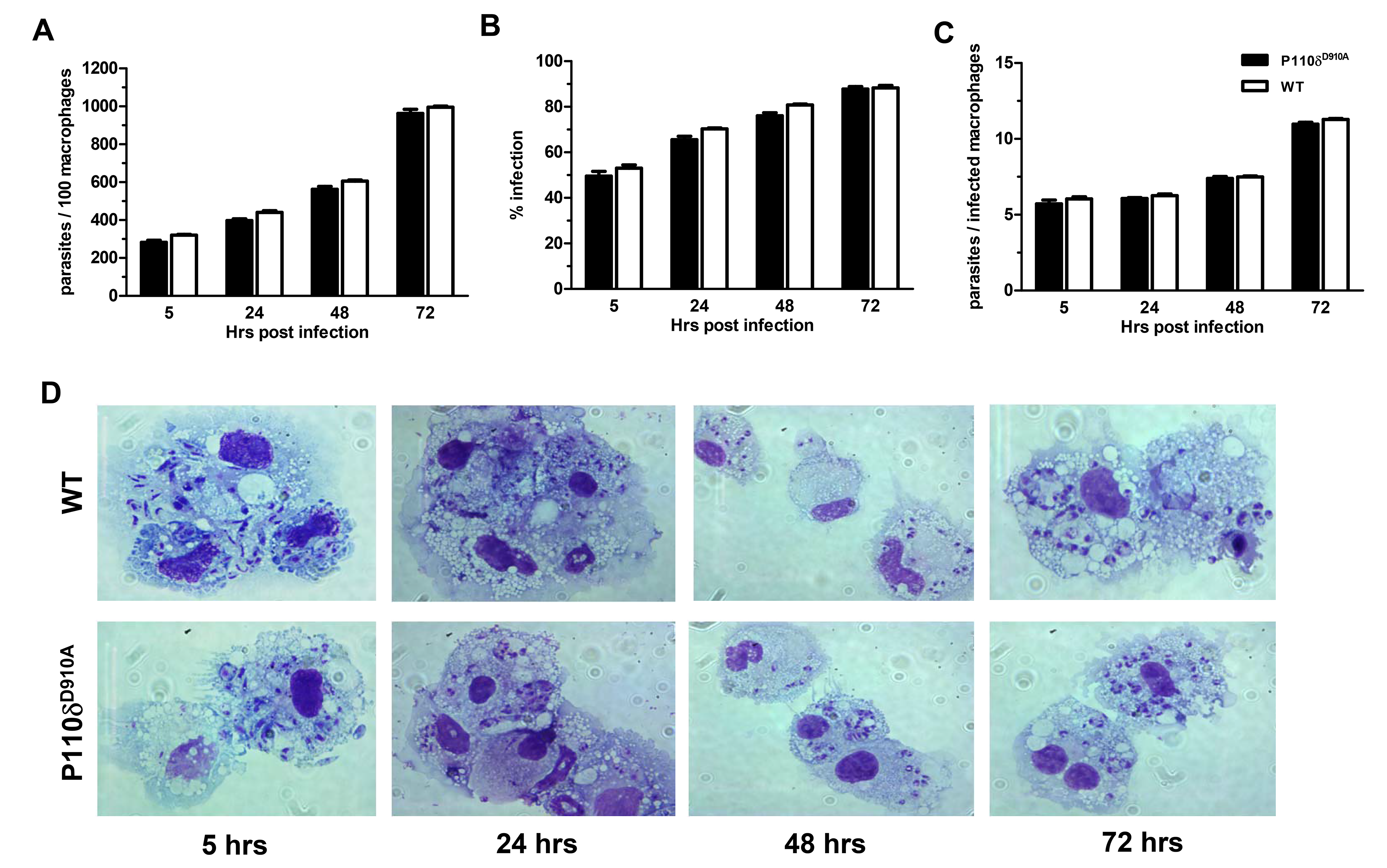

Supplement: Figure S2 — Enhanced resistance of p110δD910A mice to L. donovani is not due to superior macrophage responsiveness. Bone marrow-derived macrophages from WT and p110δD910A mice were infected with L. donovani promastigotes at a cell-to-parasite ratio of 1:5. After 24, 48 and 72 hrs, cytospin preparations were made, stained with Wright-Giemsa stain and the number of parasites per 100 macrophages (A), percent infectivity (B) and number of parasites per infected macrophages (C) were determined. (D) Light microscopy images (at ×100 (oil) objective) of infected macrophages in different time points. Results are representative of 2 independent experiments (n = 3 mice per group) with similar results. (TIF) [file pntd.0002951.s002.tif]

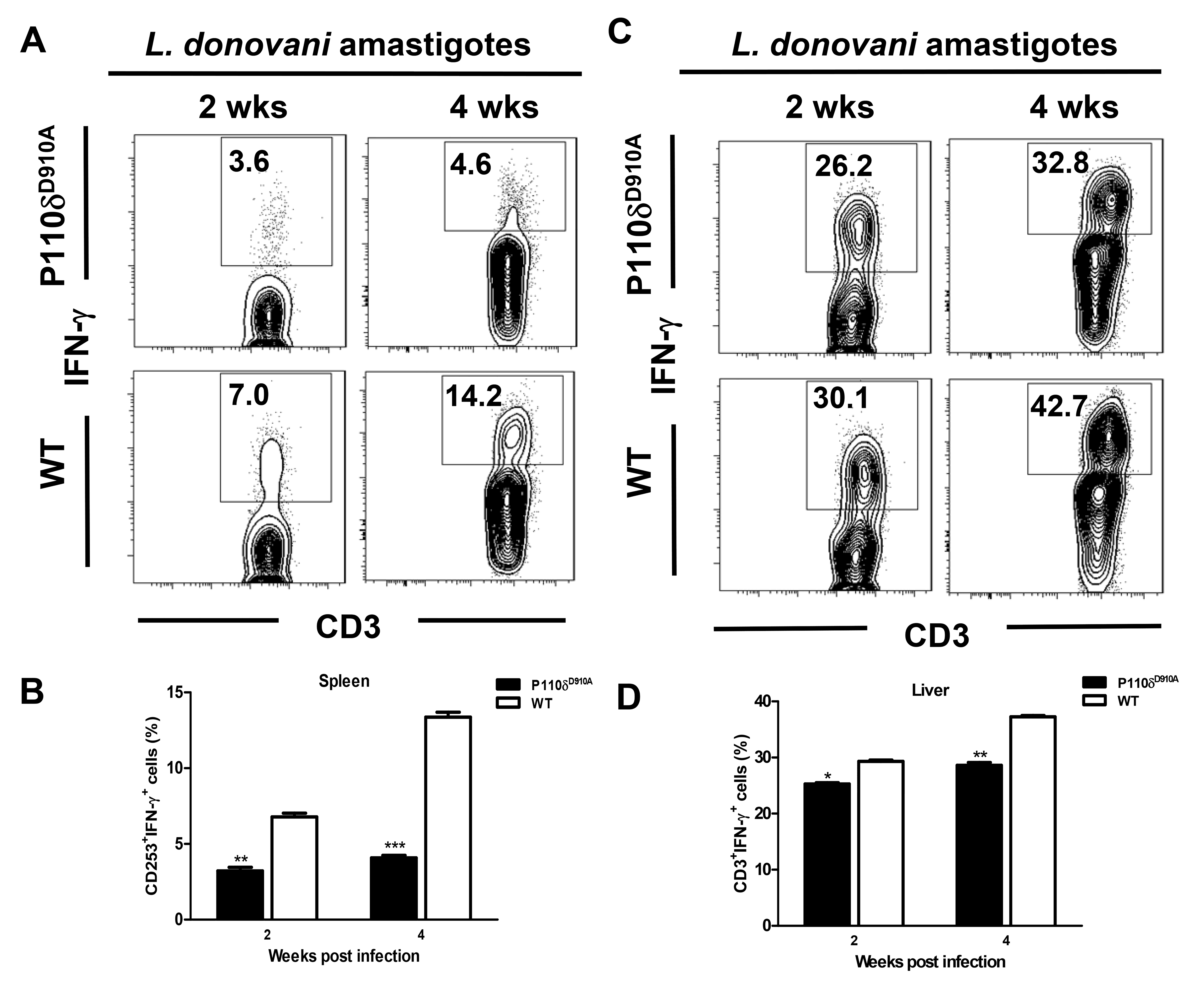

Supplement: Figure S3 — Spleen and liver lymphocytes from infected resistant p110δD910A mice produce less IFN-γ than those from WT mice. Spleen (A and B) and liver (C and D) lymphocytes from WT and p110δD910A mice infected with L. donovani amastigotes were assessed directly ex vivo at 2 and 4 weeks post infection for IFN-γ production by flow cytometry. Results are representative of 2 independent experiments (n = 3 mice per group) with similar results. Error bars, +/− SEM; *, p < 0.05; **, p < 0.01; ***, p < 0.001. (TIF) [file pntd.0002951.s003.tif]

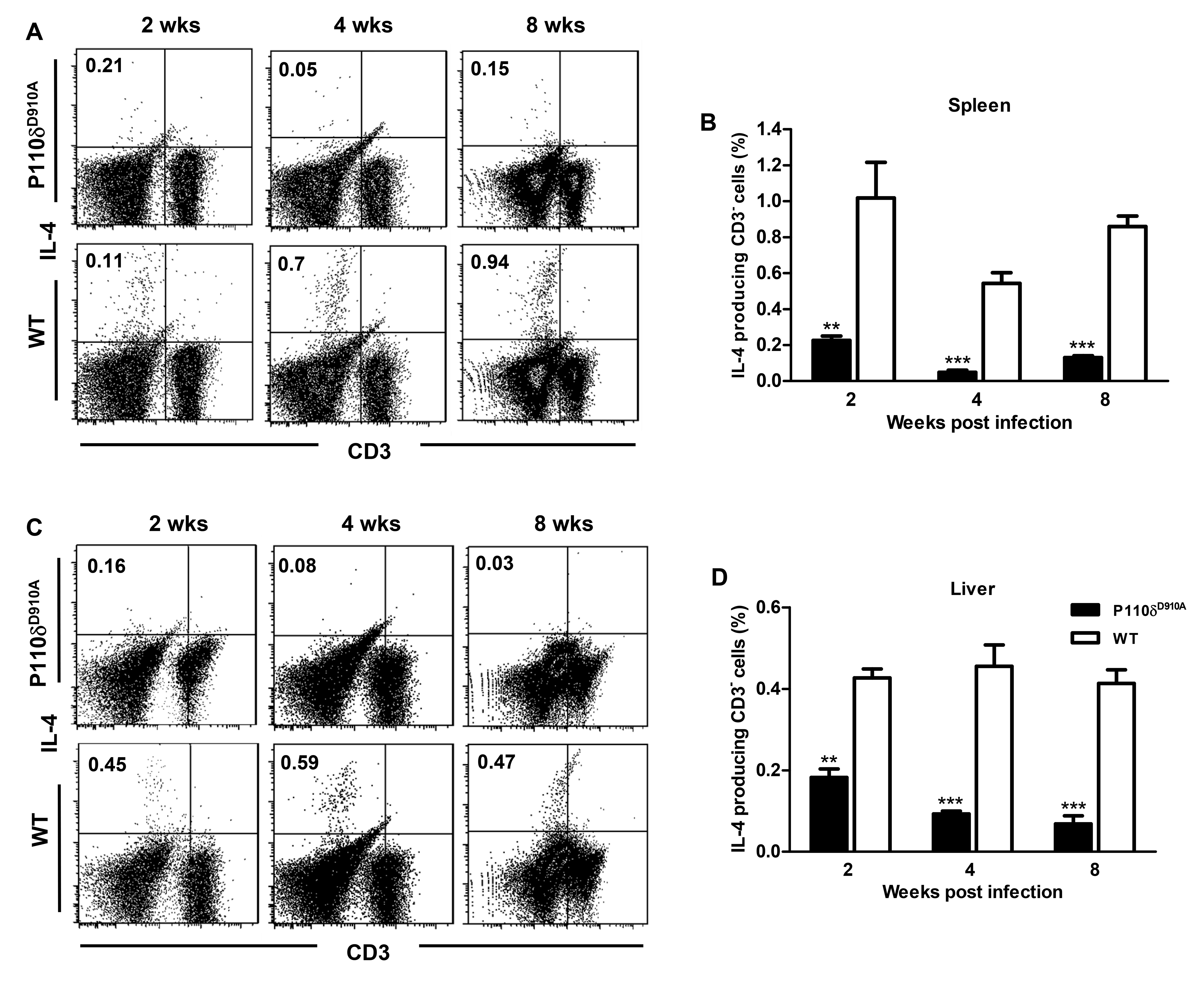

Supplement: Figure S4 — Non T cells (CD3−) are the major IL-4-producing cells in the spleens and liver of L. donovani infected WT and resistant p110δD910A mice. L. donovani promastigote infected p110δD910A and WT mice were sacrificed at the indicated times and their spleen (A, B) and liver (C, D) lymphocytes were pulsed with PMA, ionomycin and brefeldin A (BFA) for 4 hrs and directly stained ex vivo for CD3, CD4 and IL-4. Results are representative of 3 independent experiments (n = 3 mice per group) with similar results. Error bars, +/− SEM; *, p < 0.05; **, p < 0.01; ***, p < 0.001. (TIF) [file pntd.0002951.s004.tif]

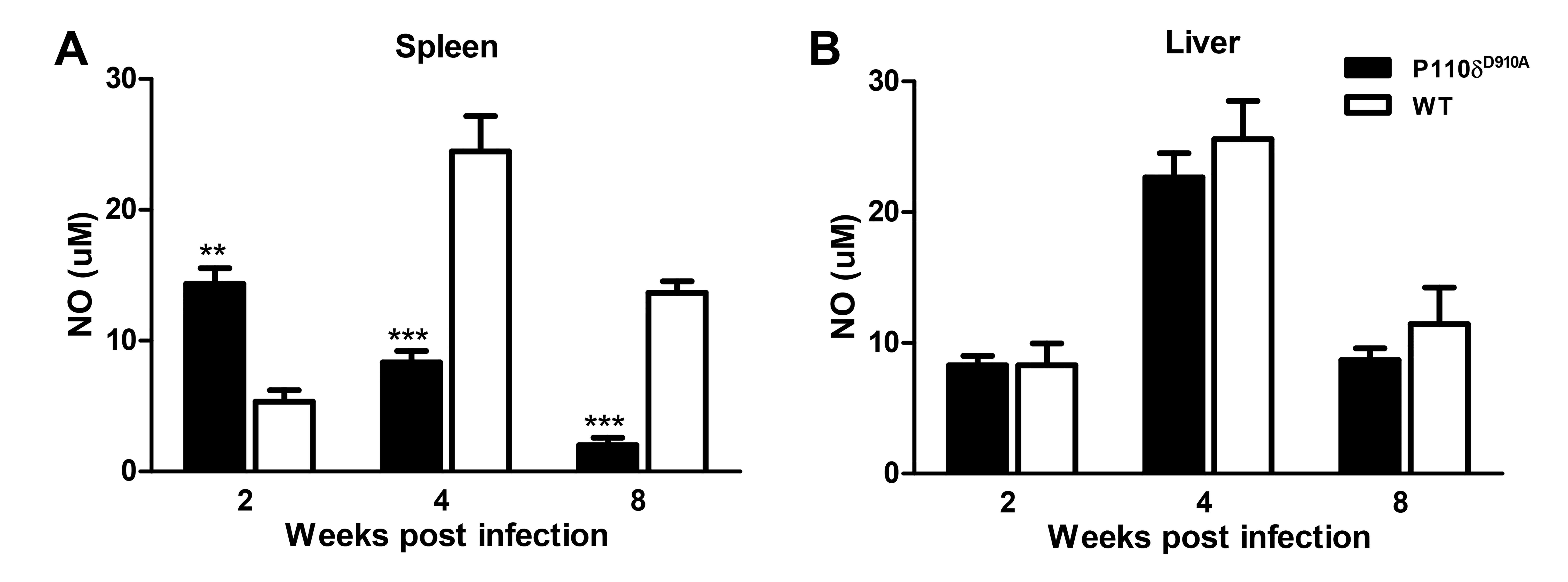

Supplement: Figure S5 — Enhanced resistance of p110δD910A mice to L. donovani is not associated with high nitric oxide (NO) production. NO levels were measured in 72 hr culture supernatant fluids of spleen (A) and liver (B) lymphocytes of L. donovani-infected WT and p110δD910A mice that were stimulated with freeze-thawed L. donovani. Results are representative of 3 independent experiments (n = 3 mice per group) with similar results. Error bars, +/− SEM; *, p < 0.05; **, p < 0.01; ***, p < 0.001. (TIF) [file pntd.0002951.s005.tif]
